# Accelerometric Gait Analysis Devices in Children—Will They Accept Them? Results From the AVAPed Study

**DOI:** 10.3389/fped.2020.574443

**Published:** 2021-01-28

**Authors:** Isabella Wiedmann, Marcello Grassi, Ibrahim Duran, Ricardo Lavrador, Evelyn Alberg, Martin Daumer, Eckhard Schoenau, Jörn Rittweger

**Affiliations:** ^1^Center of Prevention and Rehabilitation, University of Cologne, Cologne, Germany; ^2^Department of Muscle and Bone Metabolism, German Aerospace Center, Institute of Aerospace Medicine, Cologne, Germany; ^3^Department of Applied Health Science, European University of Applied Science, Brühl, Germany; ^4^Sylvia Lawry Center for Multiple Sclerosis, The Human Motion Institute, Munich, Germany; ^5^Trium Analysis Online, Munich, Germany; ^6^Technical University of Munich, Munich, Germany; ^7^Department of Pediatric and Adolescent Medicine, University of Cologne, Cologne, Germany

**Keywords:** wearables, cerebral palsy, gait speed, laboratory conditions, real-world conditions

## Abstract

**Aims:** To assess children's acceptance to wear a 3D-accelerometer which is attached to the waist under real-world conditions, and also to compare gait speed during supervised testing with the non-supervised gait speed in every-day life.

**Methods:** In a controlled observational, cross sectional study thirty subjects with cerebral palsy (CP), with level I&II of the Gross Motor Function Classification System (GMFCS) and 30 healthy control children (Ctrl), aged 3–12 years, were asked to perform a 1-min-walking test (1 mwt) under laboratory conditions, and to wear an accelerometric device for a 1-week wearing home measurement (1 WHM). Acceptance was measured *via* wearing time, and by a questionnaire in which subjects rated restrictions in their daily living and wearing comfort. In addition, validity of 3D-accelerometric gait speed was checked through gold standard assessment of gait speed with a mobile perambulator.

**Results:** Wearing time amounted to 10.3 (SD 3.4) hours per day, which was comparable between groups (*T* = 1.10, *P* = 0.3). Mode for wearing comfort [CP 1, Range (1,4), Ctrl 1, Range (1,6)] and restriction of daily living [CP 1, Range (1,3), Ctrl 1, Range (1,4)] was comparable between groups. Under laboratory conditions, Ctrl walked faster in the 1 mwt than CP (Ctrl 1.72 ± 0.29 m/s, CP 1.48 ± 0.41 m/s, *P* = 0.018). Similarly, a statistically significant difference was found when comparing real-world walking speed and laboratory walking speed (CP: 1 mwt 1.48 ± 0.41 m/s, 1 WHM 0.89 ± 0.09 m/s, *P* = 0.012; Ctrl: 1mwt 1.72 ± 0.29, 1 WHM 0.97 ± 0.06, *P* < 0.001).

**Conclusion:** 3D-accelerometry is well-enough accepted in a pediatric population of patients with CP and a Ctrl group to allow valid assessments. Assessment outside the laboratory environment yields information about real world activity that was not captured by routine clinical tests. This suggests that assessment of habitual activities by wearable devices reflects the functioning of children in their home environment. This novel information constitutes an important goal for rehabilitation medicine. The study is registered at the German Register of Clinical Trials with the title “Acceptance and Validity of 3D Accelerometric Gait Analysis in Pediatric Patients” (AVAPed; DRKS00011919).

## Introduction

Successful rehabilitation enables patients to perform activities of daily living (ADL) in their own home setting. Therefore, to monitor the success of rehabilitation will ultimately require assessments in the patient's home setting. In this respect, there is an obvious knowledge gap, as rehabilitation success is typically assessed in a clinical setting, which can only indirectly reflect the patients' functioning in their free-living environment (1). This could lead to the result that patients are prepared to pass clinical assessments but fail in their ADL. Smart wearable devices present an appealing way to circumvent this.

Within the last decade, it has become possible to assess patients in their free-living environment (2). Such wearable devices have great potential for medicine, and assessment of physical activity through commercial companies is already widespread in the public domain (3). In the adult population, accelerometric devices are being used to attempt the assessment of, for example, daily physical activity, as a surrogate for the bones' mechanical environment (4), and as a predictor of hospitalization and mortality (5). Notably, accelerometric data can nowadays also be used to accurately derive real-world gait speed, walking distance (6) and walking and running activity (7), and if worn at the wrist they are well-tolerated in healthy infant population (8).

Children often have more difficulties in following verbal test instructions (9), even if they are as simple as to walk as fast as possible. It is therefore advisable in pediatrics to acquire information in an intuitive or implicit way. In the context of gait analysis, this could, for example, occur through 3D-accelerometric assessments of gait speed, as this would only require acceptance of a measurement device, but not adherence to a specific walking test. In previous studies, it was demonstrated that wearable gait analysis devices work not only in adults, but also in children (8, 10), at least as far as afixment of recording boxes is concerned, as well as obtaining readings of gait speed that are apparently meaningful. Hence, it seems very promising in pediatrics to expand functional assessments from laboratory settings to the real world. However, the crucial question is whether children would accept such measurements.

Cerebral palsy (CP) is the most common cause of impairment in children world-wide with an incidence of 2–3 out of 1,000 live births (11, 12). CP comprises a heterogeneous etiological group, that is often associated with permanent functional deficits, and with impaired development of movement and posture (13). The neuromotor deficits “are often accompanied by disturbances of sensation, perception, cognition, communication, and behavior”(13), which introduce additional problems for testing physical functions in this patient group.

Rehabilitation of patients with CP at an early stage is paramount to the development of muscle and bone strength in early life, and thus for a healthy skeleton in the adult lifespan (14). One must also bear in mind that improving motor skills in children with cerebral palsy underpins cognitive development, non-verbal intelligence, word decoding and arithmetic function (15, 16).

Therefore, we were interested whether accelerometric data could also be collected in the assessment of children with and without CP. This question implies whether healthy children and pediatric patients and their parents would tolerate this type of measurement, and whether meaningful data can be collected. To have this information would not only be beneficial in CP children, for the reasons outlined above, but also in a wider pediatric population, given that lack of physical activity has been recognized as an increasingly important problem. Thus, we ventured to perform gait speed assessments with 3D-accelerometry recording boxes during a wearing-time of 1 week in children, to assess the children's acceptance of this novel approach (primary aim), to validate 3D-accelerometric assessment against a gold standard method, and to explore whether the laboratory-based assessment of maximal gait speed is related to gait speed in the children's habitual environment. No specific hypotheses were made *a priori* regarding any group difference in acceptance of 3D-accelerometric measurements.

## Subjects and Methods

### Participants

Sixty participants aged 3–12 years were recruited between May and October 2018 for this controlled, monocentric, not randomized, observational, cross sectional study. Thirty children with CP were recruited among patients of our rehabilitation clinic, and 30 able-bodied control children (Ctrl) were recruited from CP-participants' and from hospital staff's families. Groups were age-matched through continuously monitoring anthropometric data throughout the recruitment process. One of the inclusion criteria for CP children was, amongst others, classification with Gross Motor Function Classification Scale (GMFCS) level I and II (full list given in Table 1). CP children were excluded from study participation when they had a vagus stimulator or a ventro-peritoneal shunt. Given that no prior experience existed, no formal sample size estimation was performed, and a number of 30 children per group was deemed as (a) small enough to be feasible with existing resources, and (b) as large enough to assess effect sizes on the planned endpoints. The study flow is reflected in **Figure 3**.

**Table 1:** Inclusion and exclusion criteria.

	**Inclusion criteria**	**Exclusion criteria**
CP- group	-age 3–12 years -gross motor function classification scale (GMFCS) I&II -Patient of the Queen-Rania-Rehabilitation Center for Children, Center of Prevention and Rehabilitation, University of Cologne, Cologne, Germany -willingly to participate	-Vagus stimulator -ventriculo-peritoneal shunt
Control group	-age 3–12 years -healthy brother or sister of an included cerebral palsy (CP) child -related to a member of the staff of the Cologne Children's Hospital, University of Cologne, Cologne, Germany or of the Center of Prevention and Rehabilitation, University of Cologne, Cologne, Germany	-disability to appear for the baseline evaluation at the Queen-Rania-Rehabilitation for Children, Center of Prevention and Rehabilitation, University of Cologne, Cologne, Germany

All participants gave their written informed consent before study inclusion, where needed with support from their parents or through their parents. The study had been approved by the responsible local Ethical Committee, and it complied with the declaration of Helsinki. Before the study was commenced, it had been registered with the German clinical trials register (registration number DRKS00011919).

### Study Flow and Data Acquisition

The primary aim of the study was to assess the children's acceptance of a waist-borne 3D-accelerometer within their habitual environment. Acceptance was operationalized as the amount of wearing time and also *via* a questionnaire. The second aim was to compare gait speed during supervised testing with non-supervised gait speed in every-day life. In addition, we planned to explore the data acquired in this study in order to generate novel hypotheses with regards to validity.

**Figure 1:**
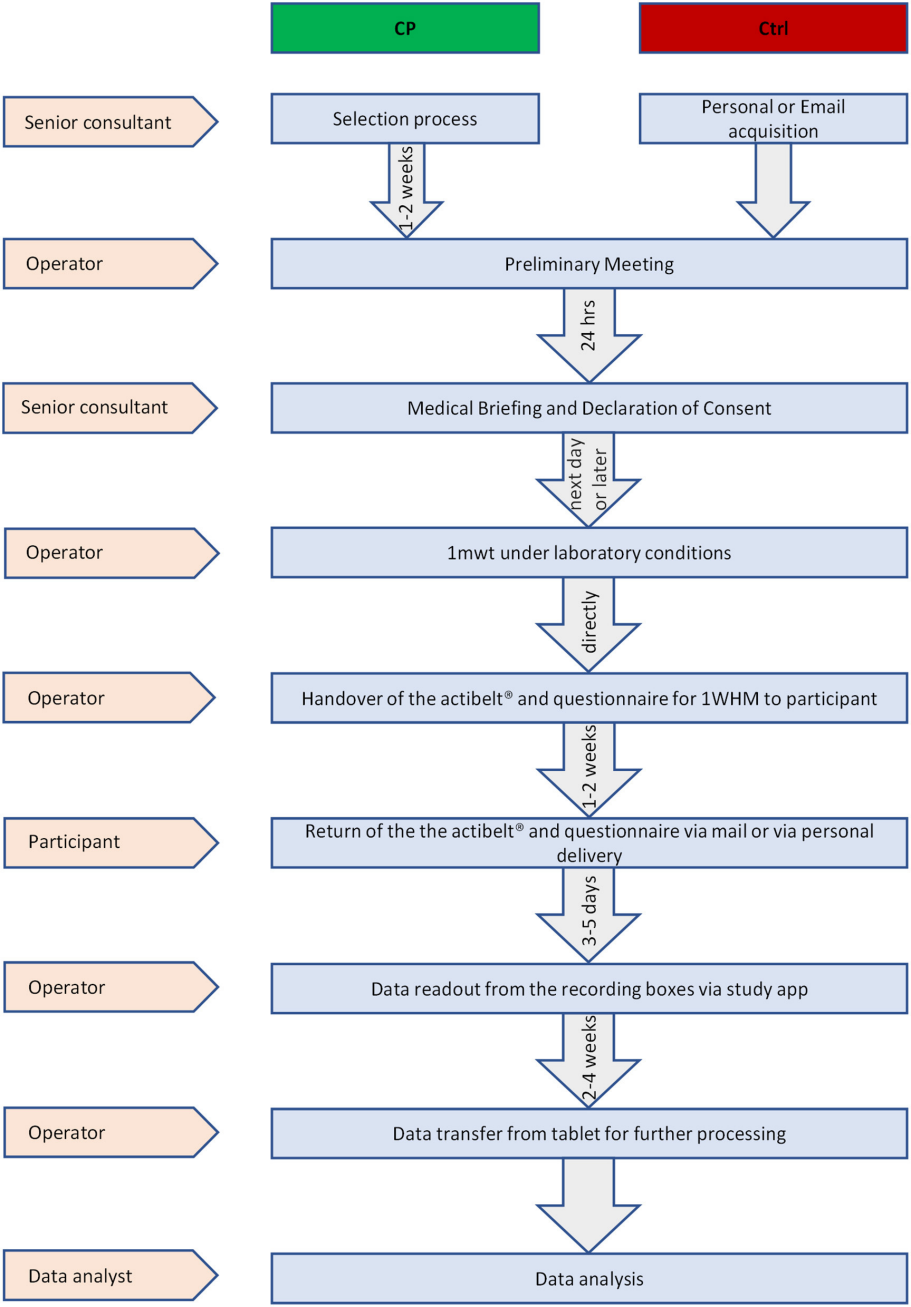
Study flow.

**Figure 2:**
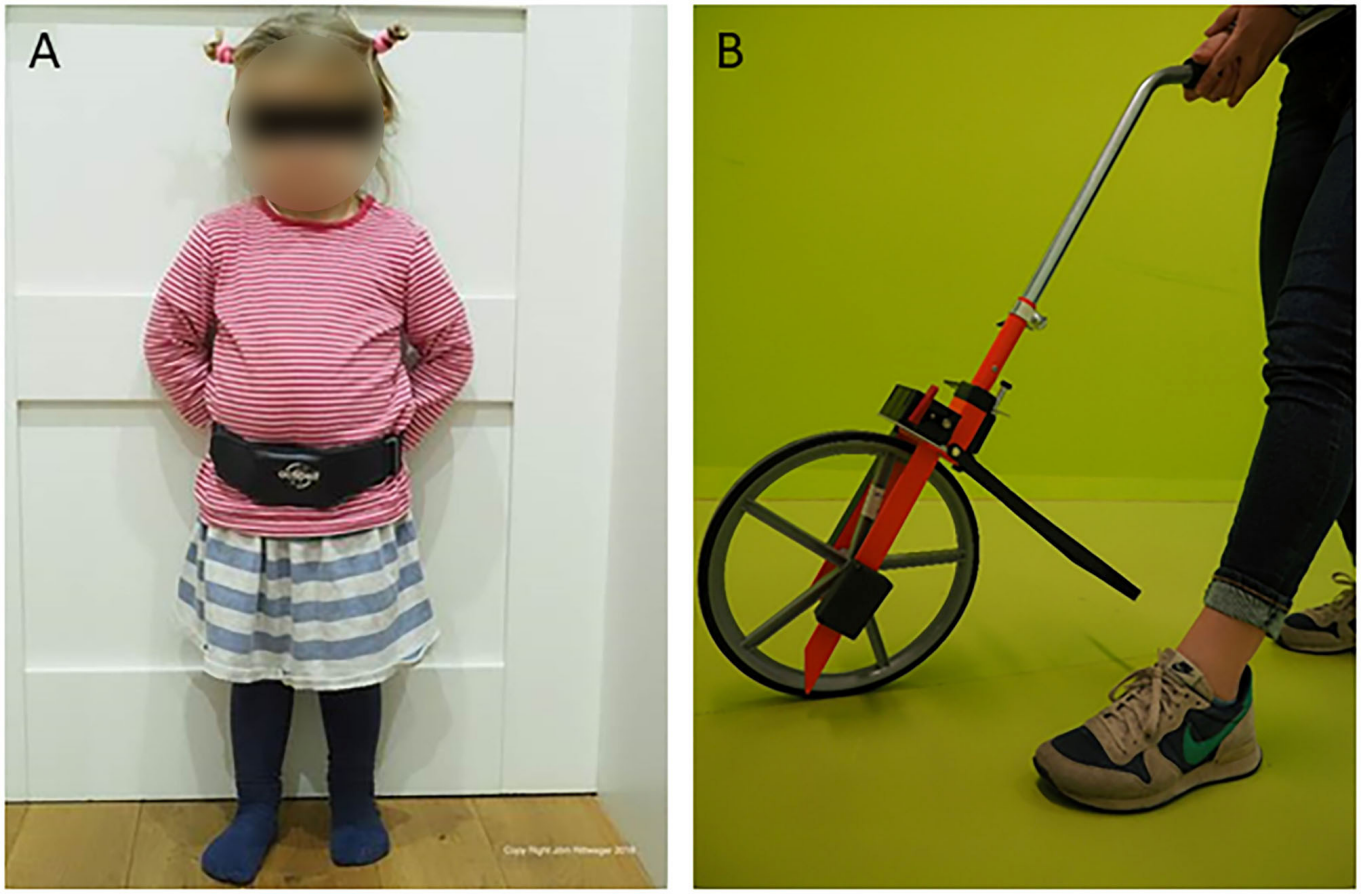
**(A)** 3-year old child wearing an actibelt®. **(B)** Mobile perambulator used in this study.

Data collection started no sooner than the day after participants had given their consent (see Figure 1). First, the 1-min-walking test (1 mwt) was performed on a standardized parkours representing “laboratory conditions.” A 3D-accelerometric recording box (actibelt®, Munich) contained in a belt buckle was worn close to the child's body center of mass in proximity of the anterior symmetry axis (Figure 2A). During the 1 mwt, one operator followed the children's course as close as possible with a mobile perambulator (Figure 2B), and the distance measured thereby served to calculate gold standard walking speed. An acoustic start signal implemented in the study tablet was provided, and the children were instructed to walk as fast as possible, whilst avoiding running, along the outlined course. After the 1 mwt, the recording box was handed over for the 1-week wearing period at home (1WHM) for measurement under “real-world conditions.” In addition to the recording box, we also handed over a questionnaire to assess wearing comfort (see Table 2). Acceptance of wearing was quantified as (a) hours of wearing per day, (b) wearing comfort as per questionnaire and (c) restriction in ADL (also per questionnaire).

**Table 2:** Questionnaire Items.

**Item**	**Scale**
Which days had the accelerometric device been worn?	Date specification
How comfortable was wearing the accelerometric device?	1 = very comfortable 6 = very uncomfortable
How much has the accelerometric device restricted the child's ADL?	1 = no restriction 6 = very restricted
Has the child refused to wear the accelerometric device partially or completely? If so, are there some reasons?	Open answers possible
Are there some ideas how to improve the device?	Open answers possible

### Material

The 3D-accelerometric device used in this study was an actibelt® RCT2 recording box. It is containing a recording box (battery capacity: 1,000 mA, battery life: >35 days, storage capacity: 4 GB, interface: USB 2.0, built in sensor: 3D accelerometer, hall effect sensor) and a wearing belt placed close to the child's center of mass. The wearing belt was equipped with a magnetic closure contact used to verify the self-reported wearing time. Three of the children had been supported with a prototype of the next generation of belts. Specifications of the 3D-accelorometric components of these new devices are compatible with the other devices used in this study.

For the assessment of the gold standard speed, the walking path of each individual child was tracked during the 1 mwt wth mobile perambulator (M10, Geofennel, Baunatal, Germany) in order to measure the distance covered. By dividing the distance by the completion time (1 min by definition), one arrives at the gold standard speed. In order to identify discrepancies between the gold standard and gait speed as provided by the actibelt®, children also wore the actibelt® during the 1 mwt.

For the measurement protocol under laboratory conditions and data readout from the recoding boxes a tablet (Toshiba, AT10LE-A) with the corresponding study app (Trium Analysis GmbH, Munich, Germany) were used.

Subjects were weighted [standing scale, Kern, type MPB 300K100P (9V, 100 mA), Germany] without shoes in their daily clothing. Their height was measured by a mobile stadiometer, type Seca 213 (Seca, Germany).

### Statistical Methods

For statistical analysis we used SPSS Statistics 24 (17) and the R-environment in its version 3.5.1 (18).

All data analyses followed the intent–to-treat principle, given that partly missing data (e.g., due to non-compliance or technical problems) should be reflected in the final analyses. Singular imputation was used for missing data, with mean for continuous variables (wearing time) and mode for ordinal variables (comfort and restriction).

Normal distribution was tested with Shapiro-Wilks and homogeneity of variances with Levene test. Wearing comfort and restriction in ADL data were statistically analyzed with the Whitney–Mann *U* test. Wearing time was tested with unpaired *t*-test. Pearson's correlation analyses was performed to assess commonality between gait speed by actibelt® and *via* gold standard in the 1 mwt. Bland-Altman plots were used in order to compare gait speed assessment with 3D-accelerometry against the gold standard. Statistically, the Bland-Altman plot data were then assessed with linear mixed effect (LME), using R's command “lme” from the package “nlme.” LME models differ from traditional analysis of variance in that normal distribution is only assumed for the residuals, but not necessarily for the data themselves. This has, for example, the advantage that LME models can deal with non-normality in body dimensions by adjustments per individual. LME-models were constructed with y-data (i.e., differences in Bland-Altman plots) as dependent variable, subject as random effect and x-data (i.e., means in Bland-Altman plots) and group (CP or Ctrl) as fixed effects. Likewise, effects of height on gait speed on Bland-Altman y-data was assessed with such LME models. To check validity of LME models, we produced residual plots and Q-Q plots and found assumptions to be valid. Furthermore, partial regression analysis was performed, using the R-package r2glmm, in order to account for the relative contribution of group, height and gait speed on discrepancies between gold standard and actibelt-assessed 1 mwt speed.

Finally, differences between groups in laboratory speed and in real-world gait speed were tested with a paired *t*-test after normal distribution and homogeneity of variances were ascertained with Shapiro's test and with F-test, respectively.

Alpha-level was set at 0,05; all variables are expressed as mean ± standard deviation.

## Results

### Recruitment Process

**Figure 3:**
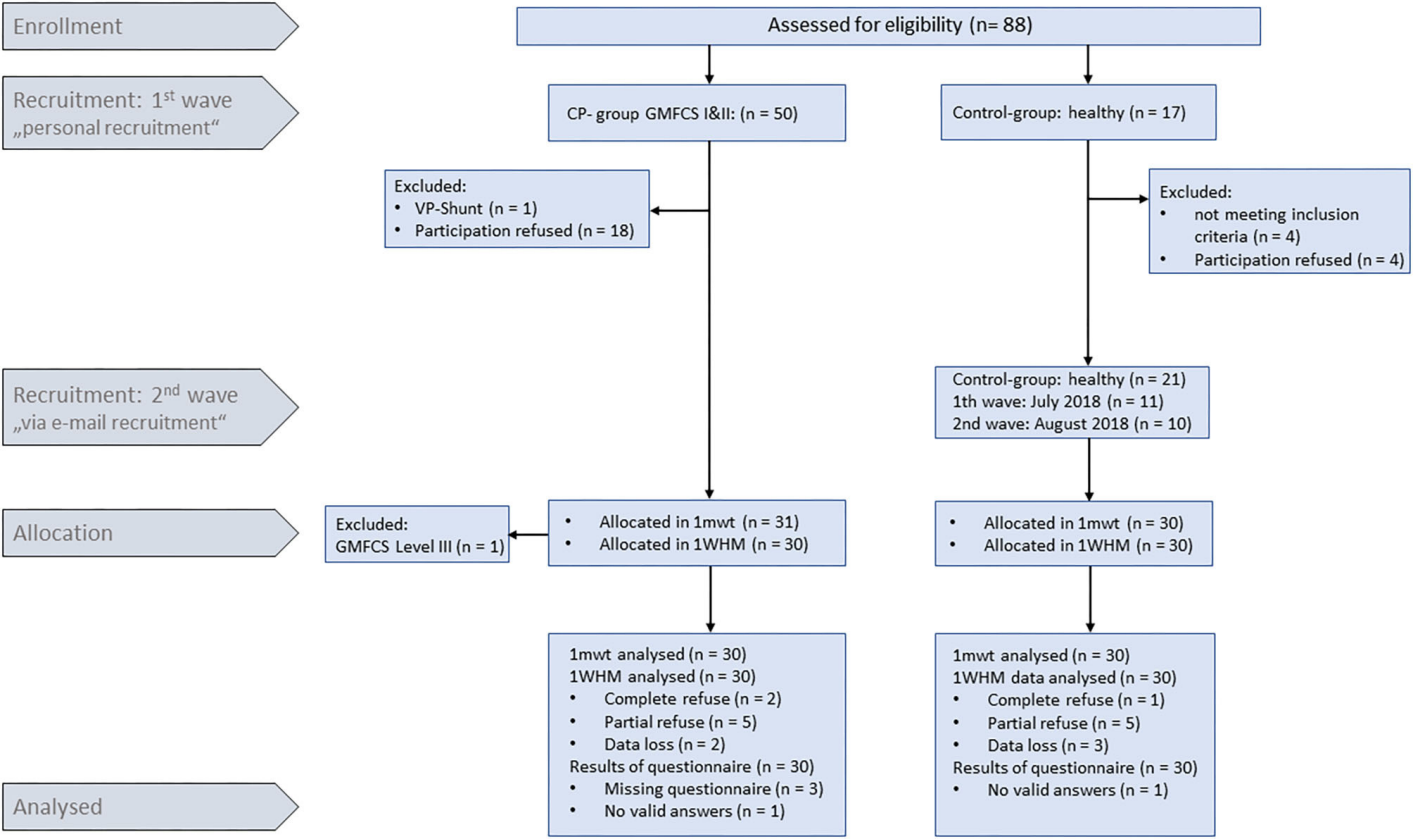
Enrolment and recruitment.

Out of 88 recruited children (67 *via* personal recruitment, 21 *via* email recruitment), 61 children performed the 1 mwt. In one child, it was found only after the 1 mwt that this child was occasionally using a walking aid, and all data were excluded from this child. In total, 21 children of the Ctrl group were recruited *via* email between July 2018 and August 2018. Thus, 60 children performed the 1 WHM (Figure 3).

Baseline characteristics of study participants (*n* = 60) are given in Table 3. Mean age ± SD in the Ctrl was 7.6 ± 3.0 years, and in the CP group 8.0 ± 3.1 years. For the Ctrl mean BMI ± SD was 16.5 ± 2.2. For the CP mean BMI was 15.7 ± 2.1.

**Table 3:** Group characteristics^a^.

	**Merged Groups**			**CP- group (*****n*** **= 30)**			**Control group (*****n*** **= 30)**			***P*-value group differences**
	**Total**	**Male**	**Female**	**Total**	**Male**	**Female**	**Total**	**Male**	**Female**	
Distri-bution	60 (100%)	36 (60%)	24 (40%)	30 (50%)	20 (33.3%)	10 (16.7%)	30 (50%)	16 (26.7%)	14 (23.3%)	
Age (years)	7.8 ± 3.0	7.6 ± 2.9	8.0 ± 3.2	8.0 ± 3.1	7.1 ± 2.9	9.7 ± 2.8	7.6 ± 3.0	8.3 ± 2.9	6.9 ± 3.1	
BMI (kg/m^2^)	16.2 ± 2.2	16.1 ± 2.2	16.1 ± 2.1	15.7 ± 2.1	16.6 ± 2.1	16.0 ± 2.1	16.5 ± 2.2	16.8 ± 2.2	16.2 ± 2.1	
Comfort of wearing mode (range)	1 (1,6)	1 (1,4)	1 (1,6)	1 (1,4)	1 (1,3)	1 (1,4)	1 (1,6)	1 (1,4)	1 (1,6)	
Restriction of daily living mode (range)	1 (1,4)	1 (1,3)	1 (1,4)	1 (1,3)	1 (1,3)	1 (1,3)	1 (1,4)	1 (1,3)	1 (1,4)	
1 mwt gait speed (m/s)	1.42 ± 0.6	1.4 ± 0.7	1.5 ± 0.3	1.3 ± 0.3	1.3 ± 0.3	1.2 ± 0.3	1.6 ± 0.3	1.6 ± 0.3	1.6 ± 0.4	<0.001
Average 1 WHM gait speed (m/s)	0.75 ± 0.4	0.69 ± 0.3	0.83 ± 0.5	0.64 ± 0.3	0.62 ± 0.3	0.66 ± 0.4	0.86 ± 0.3	0.79 ± 0.2	0.94 ± 0.4	<0.001
Maximum 1 WHM gait speed (m/s)	1.95 ± 0.22	1.98 ± 0.20	1.93 ± 0.24	1.87 ± 0.20	1.89 ± 0.19	1.82 ± 0.25	2.05 ± 0.18	2.08 ± 0.20	2.01 ± 0.17	0.0011
Total wearing time (hours/day)	10.3 ± 3.4	10.2 ± 3.2	10.5 ± 3.6	9.8 ± 3.4	9.6 ±3.8	10.4 ± 2.6	10.8 ± 3.2	11.0 ± 2.2	10.6 ± 4.3	0.3

a *The data contain the number (%) or mean ± SD*.

### Acceptance

**Figure 4:**
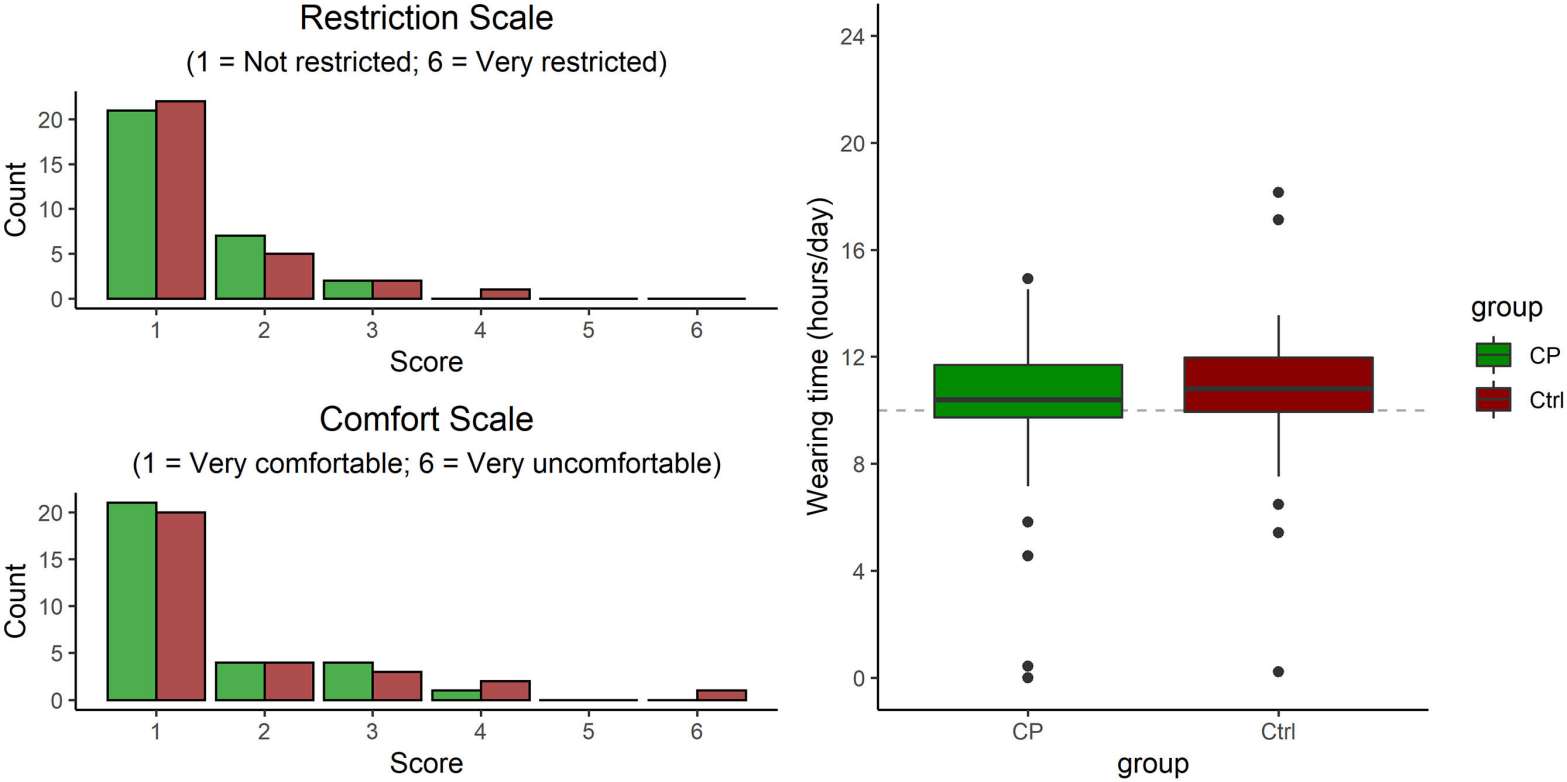
Acceptance, in terms of rating of perceived restriction and of perceived comfort (both by questionnaire) and weekly wearing time (as read from the actibelt®).

The majority of participants rated no or only little restrictions in ADL in 93.3 and 90.0% of the CP and Ctrl groups, respectively (see Figure 4, no group difference, *U* = 428, *P* = 0.9). Wearing comfort was rated as “very comfortable” in 66.6% (*n* = 20) of cases in Ctrl, and 70% (*n* = 21) in CP. Only 3.3% (*n* = 1) of participants in Ctrl scored wearing comfort as “very uncomfortable,” while none of the participants in CP scored it as “very uncomfortable” (no group difference, *U* = 441, *P* = 0.7). Wearing time amounted to 10.3 (SD 3.4) hours per day, which was comparable between groups (*T* = 1.10, *P* = 0.3).

In 8 cases we found a discrepancy between the actibelt® measured wearing time and the subject reported wearing time (5 Ctrl, 3 CP). In these cases, the recorded time from the actibelt® was used.

### Accuracy of Gait Speed With Actibelt®

**Figure 5:**
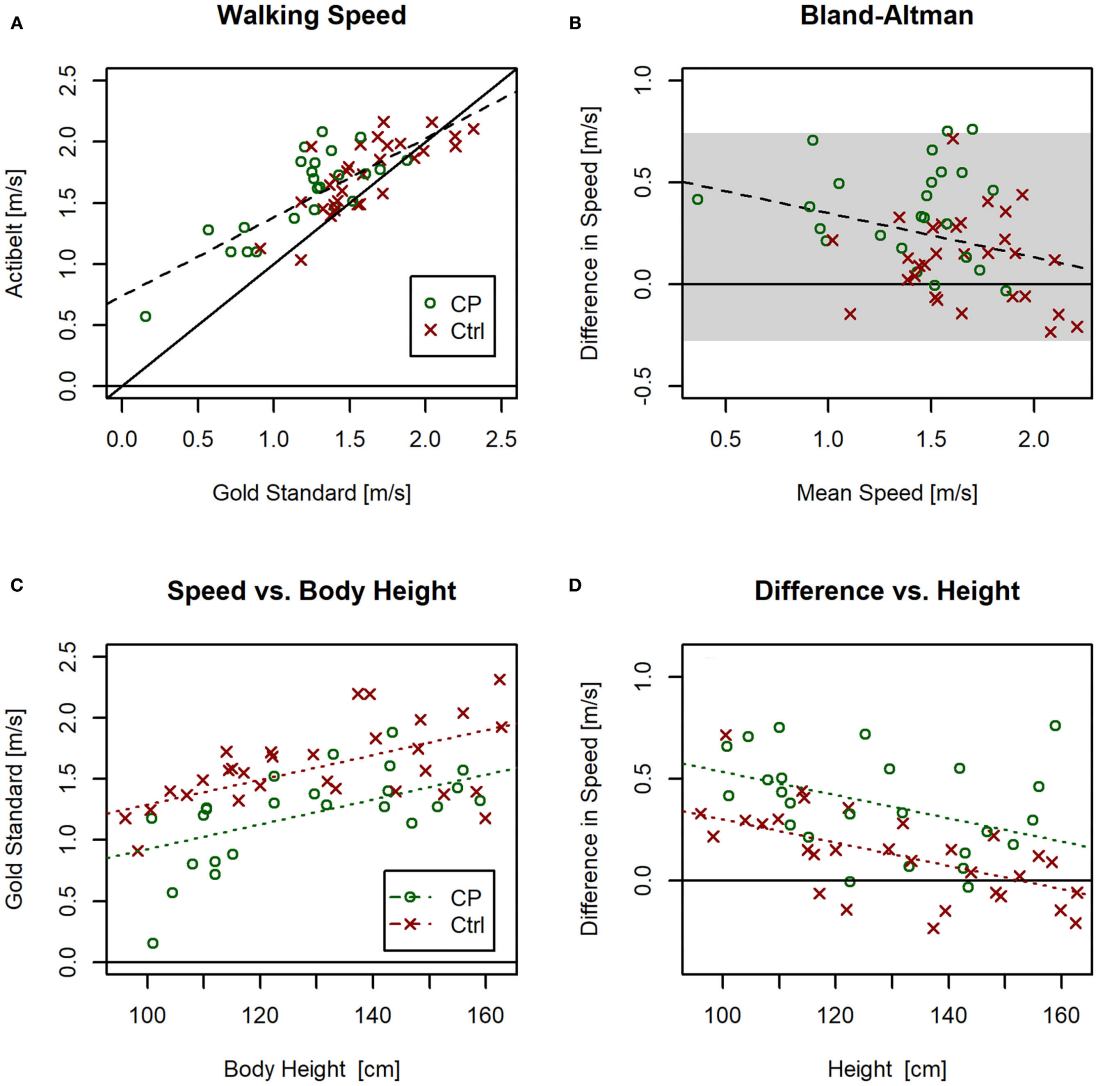
**(A)** Correlation plot between gold standard walking speed and walking speed provided by actibelt® during the 1-min walking test (1 mwt). Dashed lines represent regression lines for the CP group (blue) and the Ctrl group (green). The black solid line is the line of identity. **(B)** Bland-Altman plot for 1 mwt gait speed assessed *via* actibelt® and *via* gold standard method; the gray shaded area marks the ±2SD range of differences, and the dashed line denotes a linear relationship (*P* < 0.05). For color code refer to sub-plot A**. (C)** 1 mwt gait speed assessed by gold standard method vs. body height; significant effects of gait speed were found for group and body height, but the interaction term was non-significant. **(D)** Difference in gait speed between the two methods vs. body height; significant effects on the method-difference were found for group and body height, but not for the interaction term. For line colors refer to legend in sub-plot C.

Although there was a significant correlation between walking speed assessed with actibelt® and with the gold-standard method [Person's r = 0.78, adjusted *R*^2^ = 0.60, *F*_(1, 52)_ = 81.4, *P* < 0.001, dashed line in Figure 5A], the regression curves deviated from the line of identity [intercept = 0.74m/s as obtained from Pearson's regression, *P* < 0.001)]. When comparing both gait speed assessment methods in a Bland-Altman plot (Figure 5B), and when testing with an LME model built on those Bland-Altman data, a difference was found between the two methods [*F*_(1, 51)_ = 3,574, *P* < 0.001], suggesting that actibelt® over-estimated gait speed by 0.23 (SD 0.25) m/s. In addition, a significant linear trend [*F*_(1, 51)_ = 86.0, *P* < 0.01, see red line in Figure 5B] suggested that this over-estimation was greater at smaller gait speeds than at faster speeds. As gait speed scaled linearly with body height in both groups [Figure 5C, *F*_(1, 51)_ = 28.1, *P* < 0.001], we also explored the relationship between method-differences (= y-axis in Figure 5B) and body height. As can be seen in Figure 5D, the method-differences were inversely related to body height [*F*_(1, 51)_ = 19.6, *P* < 0.001], and regression lines were approaching 0 (meaning no difference) toward adult body height. Partial correlation analysis yielded significant contributions by group (*T* = −2.7, partial *R*^2^ = 0.13, *P* = 0.010) and by body height (*T* = −2.6, partial *R*^2^ = 0.12, *P* = 0.013), but not by gold standard speed (*T* = −1.55, partial *R*^2^ = 0.05, *P* = 0.13), indicating that accuracy of the actibelt-derived gait speed was more affected by body stature than by gait speed.

### Exploratory Analyses of Gait Speed

**Figure 6:**
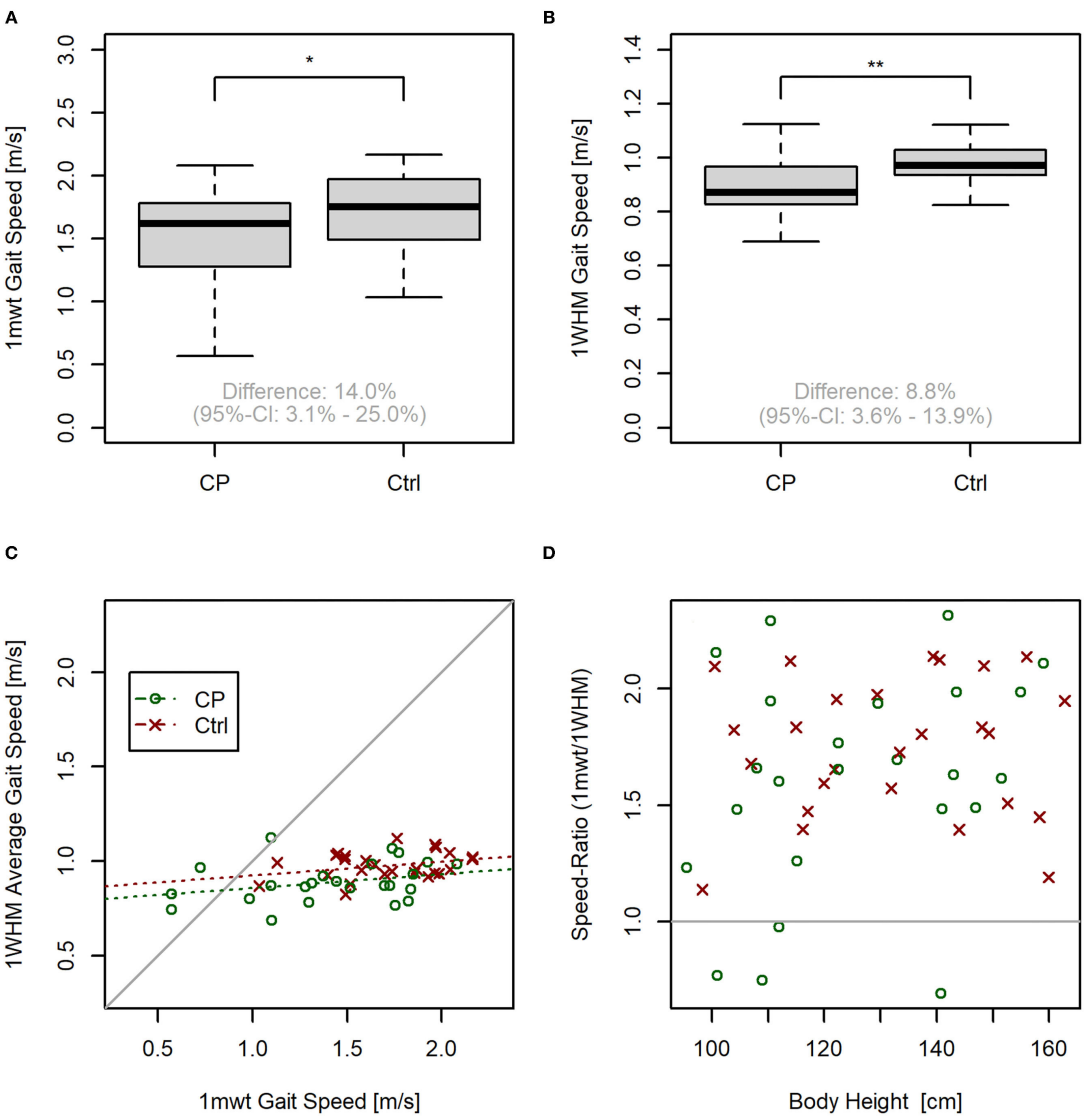
Exploratory data analysis. **(A)** Boxplot for walking speed by group in the 1-min walking test (1 mwt). **(B)** Boxplot for walking speed by group under real-world conditions. **(C)** Correlation plot between actibelt® calculated speed under real-world conditions vs. 1 mwt. Dashed lines represent regression lines for the CP and Ctrl group. **(D)** Speed ratio (obtained by dividing 1 mwt-gait speed by real-world gait speed, both assessed *via* actibelt®) vs. body height. No significant correlation was found (*P* = 0.13). *P < 0.05; **P < 0.01. For symbol colors refer to legend in sub-plot C.

When comparing 1 mwt gait speed in both groups, we found a difference between CP and Ctrl under laboratory conditions (*P* = 0.018), indicating a 14.0% (95% CI: 3.1% to 25.0% greater gait speed in Ctrl than in CP (*P* < 0.05, Figure 6A). In comparing real-world gait speed (assessed *via* actibelt®) between both groups, there was likewise a higher speed in Ctrl than in CP (*P* < 0.01, Figure 6B).

Although there is a moderate correlation between real-world gait speed and 1 mwt gait speed [*P* = 0.002, *r* = 0.26 (CP), *r* = 0,27 (Ctrl), see Figure 6C] the regression line deviated very strongly from the line of equality (intercepts 0.79 m/s and 0.85 m/s for CP and Ctrl, respectively), indicating that both variables have different information content. In addition, four children from the CP group had greater walking speed under real-world conditions than during the 1 mwt, with the latter being a maximal test. As can be seen from Figure 6D, the 1 mwt:1 WHM speed ratio was unrelated to body height (*P* = 0.13), suggesting that factors other than stature account for sub-maximal gait speed during 1 mwt in some CP children. Finally, a somewhat stronger correlation was observed between maximum 1 WHM gait speed and 1mwt speed, both obtained *via* actibelt (*P* = 0.004, *r* = 0.40).

## Discussion

Data collected from this first effort in assessing children's acceptance of 3D-gait accelerometric devices aged 3–12 years yielded very good ratings of comfort, and also low ratings for restrictions in ADL. In the past, a wearing times of 10 h per day had been regarded as the minimum, but this position has eroded and there is currently no generally accepted minimal wearing time for mobile sensor technology (19). Given that sleep is substantially longer in children than in adults, wearing times will always be shorter due to the shorter daily activity span. Results from this study demonstrate an average wearing times of 9.9 and 10.8 h in CP and control children respectively, which is considered as representative for daily activity in adults (20). This is all suggesting that children within this study have accepted wearable technology for gait speed assessment well-enough in order to provide meaningful outcomes. One could argue that CP children are already exposed to many therapeutic interventions and additional measurements. On the one hand, this could reduce hesitance to accept another device, but it could also induce aversion against yet another measurement. It has therefore been established that no group differences were found for wearing comfort and for restrictions in ADL, suggesting that children with and without a medical condition showed similar acceptance. This lets us hope that these results can be generalized to a wider pediatric population, although future studies will of course have to demonstrate this point.

In addition, many parents mostly gave positive feedback regarding easiness of use and acceptancy by their children. Negative feed-back about the device only concerned coloring (black) and material, that induced sweating during summer period.

Previous studies have almost exclusively focused on adults. Thus, a recent systematic review (21) concluded that evidence for validity and reliability for body center worn accelerometery in adults with neurological diseases is limited, and numerous conflicts in generating reliable analyses have to be solved before these devices could be used under real world conditions.

With regards to the accuracy of the gait speed measurements in the 1-min walking test, it was found that actibelt® over-estimates gait speed, as assessed with by the gold-standard method (see Figure 5B). This was evident especially at lower gait speeds, as previously also shown by Motl et.al. (22). This effect, in the population of this study, is mostly explained by variation in body height. However, it has to be noted here that existing algorithms have been validated on data from an adult population. Nevertheless, we conclude from the significant correlation between actibelt® and the gold standard method (Figure 5A) that accelerometric assessment of gait speed is principally possible also in children. One has to bear in mind here that the algorithm used to derive real-world walking speed was validated on data from an adult population (22). It therefore is possible that a validation study similar to that described by Aigner et al. (23) could remedy the problems. That this may be worthwhile is depicted in Figure 6: Gait speed differs between Ctrl and CP children both under laboratory and under real-world conditions. This was found both for the mean real-world speed as well as the maximal real-world gait speed, which confirms a recent study in patients with multiple sclerosis (24). A further look at Figure 6C suggests, that interindividual variation is lesser in the 1 WHM than in 1 mwt. We would anticipate, therefore, that within-group variation of both variables may also contain clinically meaningful information, that is, that gait speed assessment in CP children can help to judge severity of disease state and effectiveness of therapeutic strategies, or also compensatory strategies in CP children. Most importantly, assessment of real-world speed yields information that is complementary to the laboratory assessments. This is clearly evidenced by 4 children that had greater gait speed under real world conditions than when they gave their supposedly maximal effort in the 1 mwt. A trivial explanation for this surprising finding would be lack of motivation in the test. An alternative explanation would be that CP children can become over-excited during maximal efforts, and that augmented spasticity (25) during such tests limits their performance.

## Study Limitations

As with any study, there are a number of limitations. First, this had been a pilot study. Readers can now derive effect sizes from our results for their own sample size calculations. For example, for wearing time, we arrive at a sample size estimate of *n* = 99 per group to find significant group difference (α = 0.05, β = 0.2). Second, we should consider a possible selection bias. As we used two different recruitment mechanisms, that is, *via* personal contact for CP and merely *via* e-mail for Ctrl acceptance during 1 WHM is representative for both groups, but we are not able to evaluate how many healthy control children had refused participation in advance. This information, which is clearly linked to motivation, would have been helpful to judge whether the general interest in participating in a study was comparable between CP and Ctrl children. However, whilst this could have affected group differences in wearing time, we feel that such bias is unlikely to explain group differences in outcomes related to gait speed. Third, we registered the loss of two sets of data from the 1 WHM by losing the recording boxes during the reshipment process. One possible solution is to amend to the institutional address on the return envelope with a personal addressee from the research team. In having produced some invalid values in evaluation of wearing comfort and restriction of daily living we consider for future studies a more detailed written description for the use of the actibelt® and the questionnaire.

Moreover, we received feedback that recording boxes tended to slip down from their center of body mass. Thus, a solution could be to find alternatives and adaptations to the belt used by the children to position the recording box close to the body center of mass.

Another limitation regarded to the gold-standard measurement is that not having had a video recording and an accelerometer signal of the perambulator, as previously described elsewhere (23), the walking speed derived from the perambulator is given only by dividing the meters registered by the perambulator with the time to complete the test under the assumption it was always 60 s. This has some implications and limitations since it is operator-dependent and it has more room for mistakes compared to a more algorithm-based approach (accelerometer signal and video recording of toe-off/heel-strike of subjects during the test).

However, since the main objective of this study was to validate the acceptance of accelerometer in pediatric population with and without CP, more focus was given to ensuring a good compliance with the usage of the accelerometer in home environment.

## Conclusion

In conclusion, this study has demonstrated that wearable accelerometric technology can be accepted also in a pediatric population.

Based on the discrepancy of the data we received from the recording boxes of the actibelt® and the reported wearing time from the questionnaire in several cases we may presume that accelerometric devices will prove as a useful assessment of the subject's behavior in real world.

However, some adjustments are probably necessary to further miniaturize hardware, and to extend software for application in children.

## Suppliers' List

Actibelt®: The actibelt platform is a joint development by Trium Analysis Online GmbH (Munich, Germany, www.trium.de) and the SLCMSR e. V. The Human Motion Institute (www.thehumanmotioninstitute.org).

Mobile perambulator: Geofennel, Baunatal, Germany.

Standing scale: Kern, Germany.

Stadiometer: Seca, Germany.

Study app: Trium Analysis GmbH, Munich, Germany.

Study tablet: Toshiba AT10LE, Tokyo, Japan.

## Data Availability Statement

The raw data supporting the conclusions of this article will be made available by the authors, without undue reservation.

## Ethics Statement

The studies involving human participants were reviewed and approved by Ethical Committee of the University of Cologne (ID 17-184). Written informed consent to participate in this study was provided by the participants' legal guardian/next of kin.

## Author Contributions

MD, ES, and JR: conception of study. JR and ID: obtaining ethical approval. JR: study registration and running head. MG and ID: study implementation. IW, RL, ID, EA, and JR: data collection. MG: data analysis. IW, MG, and JR: statistical analysis and drafting manuscript. IW, MG, MD, ES, ID, and JR: interpretation. All co-authors: preparing and discussing final version of manuscript.

## Conflict of Interest

MG was employed by Trium Analysis Online GmbH and Sylvia Lawry Centre for Multiple Sclerosis Research e.V. at the time of the study. MD was employed by Trium Analysis Online GmbH. Trium Analysis Online GmbH, Sylvia Lawry Centre for Multiple and MD are owners of trademarks/design/patent/patent applications linked to actibelt technology (detailed list available upon request). The remaining authors declare that the research was conducted in the absence of any commercial or financial relationships that could be construed as a potential conflict of interest.

## Abbreviations

1 mwt: 1-minute-walking test
1 WHM: 1-week wearing home measurement
ADL: activities of daily living
CP: cerebral palsy
Ctrl: control children
GMFCS: gross motor function classification scale
LME: linear mixed effect model.
